# The Prevalence of Stressful Life Events among a Racially Diverse National Sample of Adolescents

**DOI:** 10.21203/rs.3.rs-8414362/v1

**Published:** 2026-01-21

**Authors:** Antonio Garcia, Kaitlyn Lovins, Nicole Karcher

**Affiliations:** North Carolina State University; University of Kentucky; Washington University in St. Louis

**Keywords:** Trauma, adolescents, discrimination, financial adversity, Adolescent Brain Cognitive Development Study^®^, BIPOC

## Abstract

**Purpose:**

Stressful life events (SLEs), such as adverse life events (ALEs), discrimination, and financial adversity, continue to be a public health concern. Limited understanding of rates of SLEs within intersecting identities hinders the implementation of interventions to reduce racial and gender disparities.

**Methods:**

Secondary analyses of data collected from 11,875 youth for the longitudinal Adolescent Brain Cognitive Development Study^®^ were conducted to examine exposure to SLEs. Raw and weighted prevalence rates of SLEs were examined by race/ethnicity, and within-group variations were examined by gender and by youth versus caregiver report.

**Results:**

Over 9% of youth endorsed experiences of discrimination (EOD). While 81.6% of youth experienced ALEs as negative, only 48.45% of caregivers did. 18.57% of caregivers reported at least one item of financial adversity. In general, Black youth experienced more SLEs, Asian youth experienced fewer SLEs, with Hispanic and Multiracial/Multiethnic youth experiencing more EOD, financial adversity, and caregiver-reported ALEs than White and Asian youth, albeit not as much as the Black racial/ethnic group. Females reported more experiences of discrimination than males.

**Discussion:**

Reporting varied by caregiver vs. youth, race, and gender. Findings call for implementing screening for SLEs, identifying strategies for reducing SLEs among Black adolescents, and training mental health professionals on screening protocol and the dynamics of intersectionality. Additional research is needed to explore cultural variations in how different groups process SLEs.

## Introduction

Adverse childhood events (ACEs) remain a public health concern, given the ongoing pervasiveness of exposures. In the original ACE study, Felitti and colleagues (1998) surveyed over 17,000 adults who received Kaiser Permanente health maintenance care about their exposures to several SLEs (previously termed adverse childhood events or adverse life events), including neglect, physical abuse, divorce, interpersonal violence, and caregiver substance abuse from 1995-1997. Since then, decades of studies have documented a dose-response relationship between ACEs and poor health and psychosocial outcomes, including but not limited to early mortality, economic hardship, psychopathology, substance use, risky sexual behaviors, and suicidal ideation (Bick & Nelson, 2016; Centers for Disease Control and Prevention, 2016; Gilbert et al., 2015; Swedo, Pampati, et al., 2024).

More recently, there has been a wave of efforts to examine the prevalence of ACEs by race/ethnicity among adult populations (Madigan et al., 2023). For example, researchers using data from the Behavioral Risk Factor Surveillance System (BRFSS) found that Black and Hispanic adults reported more ACEs compared to their White sample (Merrick et al., 2018) while respondents who identified as Multiracial had significantly elevated ACEs than all other racial/ethnic groups (Giano et al., 2020). Inequitable access to services and resources to mitigate increased exposure to poverty, economic stress, and other psychosocial conditions could explain these racial variations (Font & Maguire-Jack, 2016; Merrick et al., 2018).

Acknowledging potential recall bias related to asking adults to recall their childhood exposures, more recent studies focus on reporting during adolescence and via multiple informants across studies (e.g., caregivers, youth, and physicians [Balistreri & Alvira-Hammond, 2016; Ndjatou et al., 2024]). Relatively few studies have explored ACEs by race/ethnicity among youth, however. One study utilized data collected from parents/guardians for the National Survey of Children’s Health (NSCH) study and found that Black youth between the ages of birth to 17 were more likely to experience at least one ACE in comparison to White children, with over 60% of Black children having one or more ACE (Sacks & Murphey, 2018). In another study that collected both physician and caregiver reports on adolescent health, Balistreri and Alvira-Hammond (2016) found that Black adolescents were more likely to experience any ACE type (68.8%) compared to White adolescents (52.4%) and Hispanic adolescents (58.2%). Even when relying upon the reporting of adolescents, these disparities remain pervasive for Black, Latinx (Meeker et al., 2021), Multi-racial, and American Indian/Alaska Native groups (Swedo, Holditch Niolon, et al., 2024).

Other researchers indicate that the original adverse childhood experience measure developed by Felitti and colleagues (1998) does not capture the vast array of stressful life events that youth (and particularly youth of color) may experience (Maguire-Jack et al., 2021; Warner et al., 2023; Zimmerman & Messner, 2013). Thus, several recent studies have expanded the definition of adversity to include exposure to community violence or unsafe neighborhoods, natural disasters, economic stressors, bullying, and discrimination, among others (Bethell et al., 2017; Garcia et al., 2017, 2025; Greeson et al., 2014). Efforts to mitigate the effects of ACEs could be derailed by not having a complete understanding of the epidemiology of SLEs, thereby sustaining poor health and psychosocial outcomes among our increasingly racially diverse population of youth. To that end, additional efforts are needed to examine the prevalence estimates of the original ACEs and these other SLEs by race/ethnicity. Such efforts could specify research priorities and provide insight into which groups would benefit from culturally tailored prevention or intervention programs.

While eliminating racial/ethnic disparities is increasingly at the forefront of NIMHD priorities, efforts to achieve this goal will be hindered without considering within-group variability. To shift away from unilateral positionalities and recognize within-group variability, Crenshaw coined the term intersectionality (Choo & Ferree, 2010; Crenshaw, 1991). The term has gained recognition in the social sciences for unpacking how multiple, intersecting identities are conveyed and honored. Some studies, for example, suggest females are more likely to experience SLEs than their male counterparts (Giano et al., 2020; Meeker et al., 2021; Swedo, Pampati, et al., 2024), although it is unknown if gender disparities prevail within different racial/ethnic groups, including those who identify as Multiracial, an often-understudied group. Addressing this question can potentially challenge the underlying assumption that sex differences are pervasive or that females are more prone to SLEs across all racial/ethnic groups, thereby improving our understanding of whether people experience SLEs differently depending on intersecting identities. Understanding rates of SLEs within intersecting identities will aid 1) in developing and implementing preventative and clinical interventions, and 2) in advocacy efforts to focus on impacted communities and strive for changes to reduce disparities, including SLEs.

From an intersectional perspective, it is also imperative to consider the interplay between caregiver perceptions and youth outcomes. That is, limited research has examined whether youth reports of SLEs differ from caregivers' reports of youth exposures. Limited studies suggest that adolescents are more likely to report SLE exposures than their caregivers (Lombardi et al., 2022; Ndjatou et al., 2024), calling into question whether caregivers are aware of or informed of such incidents. However, variations in reporting have not been examined by both race and sex at birth. Understanding the nuances through an intersectional lens could deepen our efforts to strengthen caregiver recognition and responsiveness, mitigating the effects of SLEs on youth. To advance this line of inquiry, the current study relied on data collected during the Adolescent Brain Cognitive Development (ABCD) study to address the following questions: 1) Are there differences in the prevalence of SLEs between different race/ethnic groups?, and 2) Are there differences in prevalence by sex at birth within race/ethnic groups? Analyses will also determine whether prevalence differs by reporter (adolescent vs. caregiver).

## Methods

### Participants

A sample of 11,875 individuals was obtained from the ABCD Study^®^ (Data Release 6.0), a large-scale study tracking 9- to 10-year-olds recruited from 21 research sites across the United States (Barch et al., 2018). Potential participants were excluded from overall ABCD study participation for the following reasons: child not fluent in English, major neurological disorder, gestational age less than 28 weeks or birthweight less than 1,200 grams, history of traumatic brain injury, or has a current diagnosis of schizophrenia, autism spectrum disorder (moderate, severe), mental retardation/intellectual disability, or alcohol/substance use disorder. See Table 1 for sample characteristics. Analyses analyzed 2-year follow-up data collected from July 2018 through September 2021, as this maximized the number of included stressful life event variables. Analyses occurred from March 2024 through November 2025.

### Measures

#### Independent Variables: Race/Ethnicity and Sex at Birth.

Caregivers were asked to report on race/ethnicity, which contained 5 categories: Black, Hispanic, Asian, White, and Multiracial/Multiethnic (i.e., please note, while the majority of this category is multiracial/multiethnic youth, with Native American/Alaskan Native, Native Hawaiian or Other Pacific Islander, or individuals who endorsed “Other” included). Sex at birth included whether caregivers endorsed that their youth was female or male at birth.

#### Dependent Variables: Stressful Life Events Variables

Stressful life events were examined using experiences of Discrimination (EOD), financial adversity, and adverse life experiences. See Supplemental Table 1 for individual items comprising these measures.

##### Experiences of Discrimination (EOD).

The Perceived Discrimination Scale (PDS; Phinney et al., 1998) is a 7-item youth-report questionnaire asking participants how often they experienced discrimination over the past 12 months: 1) from teachers, 2) from other adults outside the school, 3) from other students based on their ethnicity, 4) how often others behaved unfairly towards their ethnic group, 5) how often they felt not wanted in society, 6) how often they felt not accepted by other Americans, and 7) how often they felt Americans have something against them. Items are rated on a 5-point scale from ‘Almost Never’ to ‘Very Often’. For analyses, we examined the average of these EOD ratings.

##### Financial Adversity.

Financial adversity was measured as the sum of endorsements to seven questions assessing caregiver-rated financial difficulties (1 = yes, 0 = no). Financial adversity was chosen as a measure of financial hardship.

##### Adverse Life Experiences.

The PhenX Adverse Life Events scale measures 25 adverse life events (ALEs) experienced by the child (Tiet et al., 2001). The ABCD separated asked this questionnaire of the youth (youth-report) and caregiver (caregiver-report). Following the endorsement of an ALE, the child (or caregiver) is asked whether the event was positive or negative. Following previous research, we calculated the number of items judged as negative (Tiet et al., 2001). Analyses separately examined youth- and caregiver-reported negative ALEs.

### Statistical Analyses

First, we examined the prevalence of both caregiver-reported and youth-reported stressful life events by type (i.e., EOD, financial adversity, and caregiver- and youth-reported ALEs) at 2-year follow-up. We also examined prevalence weights adjusted for American Community Survey propensity scores (Heeringa & Berglund, 2020) to improve the representativeness of the prevalence estimates for the U.S. population. We also examined whether youth had ever experienced an SLE and whether they had experienced four or more SLEs, given that prior studies indicate that four or more SLEs is the threshold for elevated risk of mental health problems in youth and adult populations (Alhowaymel et al., 2023; Goldenson et al., 2021). For prevalence analyses, each index is dichotomized (0 = no endorsement, 1 = 1+ endorsement). To examine prevalence rates, we used the survey package in R, accounting for clustering by family and site. We also examined whether prevalence varied by race/ethnicity (White, Black, Hispanic, Asian, Multiracial/Multiethnic) and by sex across the entire sample and within each racial/ethnic group. Differences between groups were quantified using likelihood ratio tests. Follow-up analyses used the lme4 package (Bates et al., 2015) to conduct linear mixed effect models examining total number of endorsement of EOD, financial adversity, and caregiver- and youth-reported adverse life events, and total number of SLEs as outcomes with each of the race/ethnicity and sex at birth as predictors in separate models, with family unit and site nested as random intercepts. To examine intersectionality, we used linear mixed-effect models to examine the interaction of sex at birth by racial/ethnic group comparison (e.g., Hispanic versus other racial/ethnic groups). Models first; generalized mixed-effect models examined binary SLE outcomes, with follow-up models also examining the total number of SLEs.

## Results

**Figure 1:**
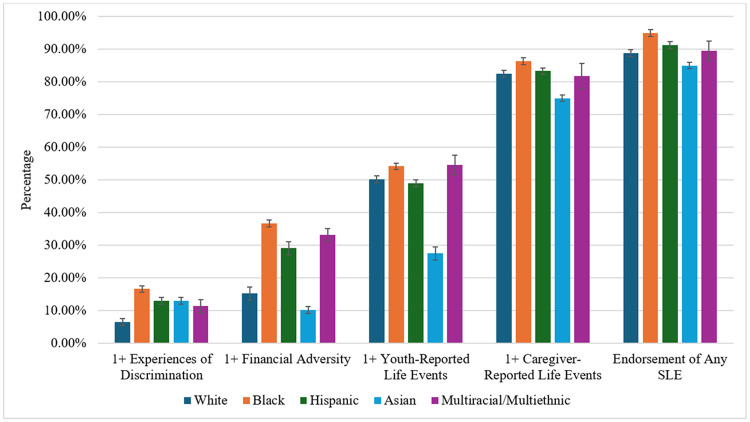
Prevalence of adjusted stressful life events (SLEs) separately by racial/ethnic group.

See Table 1 for sample characteristics. Table 2 describes the prevalence of SLEs for the full sample and for racial/ethnic and subgroups. Figure 1 provides a bar graph of SLES by race/ethnicity. Overall, there was variability in the types of stressful life events endorsed by youth and caregivers. While 9.16% of youth reported experiencing discrimination, 82.16% reported experiencing at least one adverse life event as negative. In contrast, only 48.45% of caregivers endorsed that youth experiencing an adverse life event experienced it as negative (notably, this is the same scale separately completed by either caregivers or youth; note, prevalence rates did not substantially change when including common SLEs, such as parents divorcing, youth: 82.09%, caregivers: 49.00%). 18.57% of caregivers reported at least one item of financial adversity.

### Evidence for Variation of Endorsed SLEs by Race/Ethnicity Group.

Table 2 shows variation in the prevalence of SLEs by racial/ethnic group (see Supplemental Table 2 for propensity score-adjusted prevalence rates). As seen in this table and confirmed by likelihood ratio tests (see Supplemental Table 3), across all SLEs, compared to all other racial/ethnic groups, the Asian racial/ethnic group reported lower prevalence for every SLE (*χ*^2^ ≥8.89, *p*s<.005; except for experiences of discrimination, LR=1.55, *p*=.28). Similarly, the White racial/ethnic group, compared to all other racial/ethnic groups, showed the lower rates of experiences of discrimination, financial adversity, and youth- and caregiver-reported ALEs (LRs≥7.44, *p*s<.005), although there were no significant differences for youth-reported ALEs (LR=2.28, *p*=.09).

Compared to other racial/ethnic groups, Black, Hispanic, and multiracial/multiethnic racial/ethnic groups showed the highest rates of experiences of discrimination and financial adversity (LRs≥5.37, *p*s<.05). Black and multiracial/multiethnic youth additionally showed higher rates of caregiver-reported ALEs (LRs≥5.10, *p*s<.05). Black youth additionally showed higher rates of youth-reported ALEs (LR=13.72, *p*<.001). Results remained similar when total scores for SLE measures were used as the outcome (see Supplemental Table 4).

### Intersectional Evidence for Variation of Endorsed SLEs by Race/Ethnicity and Sex.

Table 3 shows variation in the prevalence of ACEs by race/ethnicity group, separately by sex at birth (see Supplemental Table 5 for propensity score-adjusted prevalence rates; see Supplemental Table 3 for likelihood ratio tests of the effect of sex). As shown in this table, across the entire sample, females reported more experiences of discrimination than males (LR = 4.07, p < .05).

Next, we conducted models with a racial/ethnic group-by-sex-at-birth interaction to examine intersectionality. As can be seen in Supplemental Table 6, for experiences of discrimination, there was an interaction of sex at birth with Asian vs. other racial/ethnic groups, whereby for Asian youth, there was a greater effect of sex, with Asian female youth showing greater EOD than Asian male youth. For any SLE, there was an interaction of sex at birth with Black vs. other racial/ethnic groups, whereby for Black youth, there was a greater effect of sex, with Black female youth showing greater SLEs than Black male youth. When examining total scores for SLEs, there were no significant racial/ethnic group-by-sex-at-birth comparisons (see Supplemental Table 6).

## Discussion

This is the first study to explore the prevalence of SLEs with an intersectional lens. A few key findings could inform best practices and future research. First, this study suggests that SLEs, including adverse life events, EOD, and financial adversity, are pervasive among adolescents, underscoring the need to implement screening procedures to detect exposures and implement preventative interventions to overcome adverse events. Unlike other adversity-focused studies, we captured a range of SLEs typically associated with poor developmental and psychosocial outcomes, thereby providing more precise estimates of the incidence of events that affect adolescent health.

Secondly, reporting varied by source (caregiver vs. youth), race/ethnicity, and sex at birth. Indeed, findings show a stark disparity between adolescent ALEs as reported by youth versus their caregivers. Unlike previous SLE studies, a novel contribution is that youth were asked to recount their exposure to 25 events and whether they perceived each as negative (caregivers were asked the same questions about their youth). In this case, adolescents reported a higher prevalence of negative adverse life events than their caregivers did. Recent studies validating these trends suggest that caregivers may not be privy to their adolescents' experiences, may worry about the implications of reporting these exposures, or may fear the consequences of doing so. On the other hand, findings could suggest adolescents may be forthcoming about their experiences in attempts to receive help (Lombardi et al., 2022; Ndjatou et al., 2024). During ABCD data collection, caregivers grappled with remote work demands and caretaking during mandatory school shutdowns and virtual learning during the COVID-19 pandemic. Caregivers' extra demands coupled with less social support to mitigate the effects of their pre-existing conditions, such as depression and/or anxiety (Whaley & Pfefferbaum, 2023), could have impacted their ability or capacity to recognize and/or address adolescent SLEs. However, adolescents may have had varying understandings or interpretations of questions (e.g., how might they interpret “You got seriously sick”?). Regardless, findings underscore the need to ensure mental health professionals assess congruency between youth and caregiver reporting of SLEs. To that end, they may need additional training to promote effective communication about SLEs between adolescents and caregivers. With support and guidance, caregivers can play a powerful role in buffering the effects of SLEs (Racine et al., 2020; Szota et al., 2023).

Third, while our findings underscore disparities in youth and caregiver reporting, we must also call attention to racial variabilities. For example, the Black racial/ethnic group reported higher rates of EOD, ALEs, and financial adversity compared to all other groups. Hispanic and Multiracial/Multi-ethnic youth experienced elevated ALEs (per caregivers), financial adversity, and EOD than their White and Asian counterparts, while Asian youth (and their caregivers) endorsed the fewest ALEs.

In light of the findings, we can conclude that 1) Black youth experience more SLEs than other groups, 2) Asian youth experience fewer SLEs, 3) Hispanic and Multiracial/Multiethnic youth tend to experience more SLEs, including EOD, financial adversity, and caregiver-reported ALEs than White and Asian youth, albeit not as much as the Black racial/ethnic group does. While previous studies report a higher prevalence of youth-reported SLEs among Multiracial (Swedo, Holditch Niolon, et al., 2024), Black, and Latinx youth (Meeker et al., 2021), the current study extends prior work by exploring a range of SLEs. Notably, our findings show that youth-reported EODs are pervasive among youth of color, underscoring the need for mental health professionals to routinely screen for EODs, and cultivate strategies with them to navigate the challenges of discrimination and oppression. Lastly, the stark contrast between the Black group faring worse as it relates to financial adversity and the Asian group experiencing less than other groups can’t be ignored. As prior research also solidifies that Black youth are significantly more likely to experience economic hardship (McLoyd, 1990; Williams & Baker, 2021), more concerted efforts should be devoted to implementing policy packages, inclusive of expanding the earned income credit and increasing the federal minimum wage and the Supplemental Nutrition Assistance Program (Pac et al., 2023).

### Implications and Contributions

Notably, disparities at the intersections of race and sex at birth were observed. When examining sexdifferences, we found that females reported more EOD than males. However, there was an interaction of race*sex at birth under two conditions: 1) Asian female youth reported greater EOD than Asian male youth, and 2) for any SLE, Black female youth showed greater SLEs than Black male youth. In previous studies, it has been reported that while Black youth experience more discrimination than other racial groups, other children of color still experience discrimination at rates that exceed their White counterparts (Nagata et al., 2021). The current study advances our understanding of these experiences by highlighting that Asian and Black female youth experience elevated levels of adversities. Plagued by negative stereotypes, racial profiling, unequal treatment in schools and other child-serving systems of care, and daily microaggressions, children of color are primed to experience a range of negative psychosocial and health outcomes (Benner et al., 2018; Clark & Gochett, 2006). As for White youth, the effects of EOD should not be overlooked. On the contrary, females may be at heightened risk due to sexism and harassment (Lee, 2024; Stark et al., 2021). To our knowledge, this is the first study to report these findings; thus, more research is needed to validate them. Still, it raises the question of whether there are cultural, perceptual, or attitudinal variations in how different groups observe or process SLEs.

The current study advances science by examining the prevalence of exposure to SLEs beyond traditional ACE screenings and by examining differences through an intersectional lens. Still, there are limitations to acknowledge. First, this study offers a crosssectional snapshot of SLEs; thus, longitudinal research is warranted. Second, we must acknowledge that interpretations of survey questions may differ by race, sex, and reporting source (i.e., youth vs. caregiver). Despite these limitations, our findings indicate that SLEs vary not only by race/sex but also by adolescents versus caregiver reporting.

Future research is needed to examine exposure to SLEs over longer periods and identify the specific culturally applicable supports or interventions needed to mitigate the potentially harmful effects of these exposures. More immediate attention could be devoted to implementing universal screening for SLEs, identifying strategies for reducing SLEs among Black adolescents, incorporating strategies for youth of color to grapple with EODs, and training mental health professionals on the dynamics of intersectionality, and on how to cultivate open lines of communication about SLEs between adolescents and caregivers reporting and recognition of them.

## Supplementary Material

This is a list of supplementary files associated with this preprint. Click to download.

• CopyofSupplementalTablesv5final.xlsx

## Figures and Tables

**Table 1.** Sample Characteristics

	Total Sample		
	N	Mean	SD
2-Year Follow-up			
Age in Years	10973	12.07	0.67
	N	%	SE
Female	5218	47.6	0.005
White	5871	53.5	0.005
Black	1587	14.5	0.003
Hispanic	2198	20.0	0.004
Asian	209	1.9	0.001
Multiracial/Multiethnic	1106	10.1	0.003

Abbreviations. SD=standard deviation; N=sample size; %=percentage; SE= standard error

**Table 2.** Stressful Life Event Unadjusted Prevalence Rates in Whole Sample and by Race/Ethnicity Group and Sex at Birth

	Total Sample (n=10,973)		White (n=5,871)		Black (n=1,587)		Hispanic (n=2,198)		Asian (n=209)		Multiracial/ Multiethnic (n=1,106)	
Variable Label	%	SE	%	SE	%	SE	%	SE	%	SE	%	SE
Endorsement of 1 or more dichotomous Experiences of Discrimination items	9.16%	0.01	5.64%	0.01	16.78%	0.01	12.01%	0.01	11.33%	0.01	11.42%	0.01
Endorsement of Any Financial Adversity Item	18.57%	0.02	11.20%	0.01	33.95%	0.02	25.86%	0.01	8.87%	0.02	24.06%	0.04
Youth Endorsement of Any ALE Item	82.16%	0.01	81.26%	0.01	86.21%	0.01	83.50%	0.01	72.91%	0.04	80.43%	0.02
Caregiver Endorsement of Any ALE Item	48.45%	0.01	46.97%	0.01	53.85%	0.01	48.23%	0.02	26.60%	0.03	53.37%	0.02
Endorsement of Any SLE	89.28%	0.01	87.52%	0.01	94.69%	0.01	91.16%	0.01	82.27%	0.03	88.76%	0.02
Endorsement of 4+ SLE	42.97%	0.02	37.08%	0.02	57.69%	0.02	48.84%	0.02	23.15%	0.04	46.16%	0.03

**Table 3.** Stressful Life Event Undjusted Prevalence Rates in Whole Sample and by Race/Ethnicity Group Separately by Sex

	Total Sample (n=10,973)		White (n=5,871)		Black (n=1,587)		Hispanic (n=2,198)		Asian (n=209)		Multiracial/ Multiethnic (n=1,106)	
By Male Sex												
Variable Label	%	SE	%	SE	%	SE	%	SE	%	SE	%	SE
Endorsement of 1 or more dichotomous Experiences of Discrimination items	8.61%	0.01	5.45%	0.01	16.03%	0.02	11.19%	0.01	11.93%	0.03	10.31%	0.01
Endorsement of Any Financial Adversity Item	18.50%	0.02	11.91%	0.01	33.64%	0.02	24.64%	0.02	9.17%	0.03	24.13%	0.04
Caregiver Endorsement of Any ALE Item	82.63%	0.01	46.22%	0.01	56.56%	0.02	46.93%	0.02	31.19%	0.04	50.64%	0.02
Youth Endorsement of Any ALE Item	47.89%	0.01	82.18%	0.01	86.75%	0.01	82.94%	0.01	76.15%	0.04	80.11%	0.02
Endorsement of Any SLE	89.53%	0.01	87.99%	0.01	95.10%	0.01	90.88%	0.01	88.07%	0.03	88.03%	0.02
Endorsement of 4+ SLEs	43.14%	0.02	45.60%	0.01	55.23%	0.02	45.76%	0.02	31.19%	0.04	49.54%	0.02
By Female Sex												
Variable Label	%	SE	%	SE	%	SE	%	SE	%	SE	%	SE
Endorsement of 1 or more dichotomous Experiences of Discrimination items	9.76%	0.01	5.84%	0.01	17.53%	0.01	12.91%	0.01	10.64%	0.03	12.57%	0.01
Endorsement of Any Financial Adversity Item	18.65%	0.02	10.39%	0.01	34.26%	0.03	27.21%	0.02	8.51%	0.02	24.00%	0.04
Caregiver Endorsement of Any ALE Item	81.65%	0.01	47.82%	0.02	51.13%	0.02	49.65%	0.02	21.28%	0.05	56.19%	0.03
Youth Endorsement of Any ALE Item	49.06%	0.01	80.21%	0.01	85.66%	0.01	84.11%	0.01	69.15%	0.06	80.76%	0.02
Endorsement of Any ALE	89.00%	0.01	86.98%	0.01	94.29%	0.01	91.46%	0.01	75.53%	0.05	89.52%	0.02
Endorsement of 4+ SLEs	42.78%	0.02	47.04%	0.02	50.86%	0.02	48.56%	0.02	20.21%	0.06	55.05%	0.03

## Data Availability

Data used in the preparation of this article were obtained from the Adolescent Brain Cognitive Development SM (ABCD) Study ( [https://abcdstudy.org](https://abcdstudy.org) ). The data are publicly available to those who obtain a data use agreement from the NIMH Data Archive (NDA).
